# *Fusobacterium nucleatum*-Induced Impairment of Autophagic Flux Enhances the Expression of Proinflammatory Cytokines via ROS in Caco-2 Cells

**DOI:** 10.1371/journal.pone.0165701

**Published:** 2016-11-09

**Authors:** Bin Tang, Kun Wang, Yin-ping Jia, Pan Zhu, Yao Fang, Zhu-jun Zhang, Xu-hu Mao, Qian Li, Dong-Zhu Zeng

**Affiliations:** 1 Department of Clinical Microbiology and Immunology, Southwest Hospital & College of Medical Laboratory Science, Third Military Medical University, Chongqing, China; 2 Department of General Surgery and Center of Minimal Invasive Gastrointestinal Surgery, Southwest Hospital, Third Military Medical University, Chongqing, China; 3 Emei Sanatorium of PLA Rocket Force, Emeishan, China; Swedish Neuroscience Institute, UNITED STATES

## Abstract

*Fusobacterium nucleatum* (*F*. *nucleatum*) plays a critical role in gastrointestinal inflammation. However, the exact mechanism by which *F*. *nucleatum* contributes to inflammation is unclear. In the present study, it was revealed that *F*. *nucleatum* could induce the production of proinflammatory cytokines (IL-8, IL-1β and TNF-α) and reactive oxygen species (ROS) in Caco-2 colorectal) adenocarcinoma cells. Furthermore, ROS scavengers (NAC or Tiron) could decrease the production of proinflammatory cytokines during *F*. *nucleatum* infection. In addition, we observed that autophagy is impaired in Caco-2 cells after *F*. *nucleatum* infection. The production of proinflammatory cytokines and ROS induced by *F*. *nucleatum* was enhanced with either autophagy pharmacologic inhibitors (3-methyladenine, bafilomycin A1) or RNA interference in essential autophagy genes (ATG5 or ATG12) in Caco-2 cells. Taken together, these results indicate that *F*. *nucleatum*-induced impairment of autophagic flux enhances the expression of proinflammatory cytokines via ROS in Caco-2 Cells.

## Introduction

*Fusobacterium nucleatum* (*F*. *nucleatum*) is a gram-negative anaerobic bacterium that plays an etiologic role in the development of periodontitis [1], pulmonary infection [2], inflammatory bowel disease (IBD) [3] and colorectal cancer (CRC) [4–6]. *F*. *nucleatum* is a well-known pathogenic bacteria of peridontal disease, and *F*. *nucleatum* infection is also relative with gastrointestinal disease. It has been accepted that *F*. *nucleatum* infection could cause IBD, and contribute to colorectal cancinogenesis [7]. The interactions among the bacterium, intestinal epithelium and host innate defense responses are critical for the consequences of *F*. *nucleatum* infection. *F*. *nucleatum* infection plays a critical role in the development of intestine inflammation, which is known as a risk factor for colorectal carcinogenesis [8]. The inflammatory response to *F*. *nucleatum* infection is the main mediator of pathological changes in the intestine mucosa, but the regulatory mechanisms of *F*. *nucleatum*-induced inflammation are still not well understood.

Macroautophagy (hereafter autophagy), is an intracellular quality and quantity biological regulation process in which intracellular components are sequestered by phagophores and elongated into autophagosomes, and delivered to autolysosomes with lysosomes for degradation [9]. Autophagy contributes to remove both large macromolecular aggregates and defective or surplus organelles, and is also involved in clearance of intra-cellular bacteria and viruses [10]. In the presence of infection with a pathogenic microorganism, the evolving battle between the host cells and infecting microbes decides the pathogenesis and consequence of the infection, and autophagy plays a crucial role in the battle [11]. For example, numerous medically-important pathogens, such as *Francisella tularensis* [12], *Listeria monocytogenes* [13], and *group A Streptococcus* [14], are degraded *in vitro* by autophagy. However, some intracellular pathogens such as *Mycobacterium tuberculosis* [15] and Shigella [16], develop some mechanisms to resist the autophagic microbicidal defenses and subvert autophagy to survive, leading to persistent infection and inflammation.

Reactive oxygen species (ROS) are reactive molecules and free radicals derived from molecular oxygen [17]. ROS, mainly produced from the mitochondrial electron transport of aerobic respiration, have a role in regulating cell signaling pathways, including the activation of cell signaling cascades, gene expression, and apoptosis [18–19]. A recent investigation found that ROS are implicated in cellular activity to a variety of inflammatory responses [20]. In previous study, *F*. *nucleatum* greatly stimulated ROS production, which has a critical role in inducing the tissue inflammatory response of C57BL/6 mice model [21]. However, the role of ROS in *F*. *nucleatum*-induced inflammation in intestinal epithelial cell has not been explored.

To date, information on the role of autophagy and ROS in the induction and progression of *F*. *nucleatum*-induced inflammation is scarce in the literature. Therefore, we investigated whether *F*. *nucleatum* infection could impair the autophagic flux in Caco-2 cells after, and determined that the impairment of autophagic flux could enhance *F*. *nucleatum*-induced inflammation via ROS in Caco-2 cells.

## Materials and Methods

### Cell culture, Bacterial strains, and Animal experiments

Caco-2 and CW-2 cells, two of human epithelial colorectal) adenocarcinoma cells, were provided by the cell bank of Chinese Academy of Sciences, and grown in DMEM (Gibco, 11965–092) or RPMI 1640 medium (Gibco, 11875–093) containing 100 U/ml streptomycin/penicillin (Gibco, 15140–122) and 10% fetal bovine serum (Gibco, 10099–141) at 37°C in 5% CO_2_.

*F*. *nucleatum* ATCC 25586 was grown on Tryptic soy containing 5% defibrinated sheep blood at 37°C for 2 days under anaerobic conditions (10% H_2_, 5% CO_2_, and 85% N_2_) using Anoxomat^TM^ MarkⅡanaerobic gas filling system (Mart Microbiology, The Netherland). For infecting cells, colonies from the plate were suspended in 2 ml sterile PBS to OD_600nm_ = 1×10^9^ CFU/ml. The suspended *F*. *nucleatum* was centrifuged at 2500×g for 5min and then resuspended in DMEM medium without antibiotics (OD_600nm_ = 5×10^6^ CFU/ml). Bacterial solution (2ml) was used to infect Caco-2 or CW-2 cells.

6 weeks old C57BL/6 mice were subjected for a period of 5 weeks to gavage feeding of 1 ml bacterial solution in PBS (10^8^ CFU/ml) daily. Control mice were gavaged by 1 ml PBS [22]. Then, the histological analysis of mice intestinal tissue was performed with H&E staining. The study was approved by the ethics review board at Third Military Medical University.

### Reagents and Antibodies

3-methyladenine (3-MA, M9281), 4,5-dihydroxy-1,3-benzene disulfonic acid (Tiron, 172553), bafilomycin A1 (Baf A1, B1793), rapamycin (Rapa, R8781) and Thapsigargin (Thap, T9033) were purchased from Sigma-Aldrich. Antibodies against MAP1LC3B (L7543), ATG12 (WH0009140M1) or ATG5 (WH0009474M1) were obtained from Sigma, whereas antibodies against β-actin (sc-10731) and SQSTM1 (sc-28359) were purchased from Santa Cruz Biotechnology.

### Measurement of ROS Production

Intracellular ROS levels were detected with 2', 7'-dichlorofluorescein diacetate (DCFH-DA) assay. Caco-2 Cells were incubated with DCFH-DA (5 mM) for 30 min at 37°C in 5% CO_2,_ and then washed three times with Hank's balanced salt solution. FACS analysis determined intracellular ROS levels using a FACScan cytometer, and the data analysed by CellQuest software.

### siRNA Assay

Small interfering RNAs (siRNAs) specific for ATG12 (human, sc-72578), ATG5 (human, sc-41445), along with a control siRNA (sc-44230), each with 19–25 nucleotides, were obtained from Santa Cruz Biotechnology (Texas, USA). Caco-2 cells were transfected with 100 nM siRNA (ATG12 and ATG5) and 10 nM Dharmafect 1 transfection reagent (Thermo Scientific, T-2001-03) for 24 hours.

### Transmission Electron Microscopy

After *F*. *nucleatum* infection, Caco-2 cells were fixed in a solution containing 0.1% glutaraldehyde, 2% paraformaldehyde and 0.1 M sodium cacodylate for 2 hours, and fixed with 1% OsO4 for another 2 hours, washed three times with 1ml PBS and stained in 3% aqueous uranyl acetate for 1 hour. The fixed Caco-2 cells were then washed three times again, dehydrated with a graded alcohol series (40%, 50%, 70%, 80%, 90% and 100%), and finally embedded in Epon-Araldite resin (Canemco, #034). Ultrathin sections were cut with a Reichert ultramicrotome, counterstained with 0.3% lead citrate and examined on a Philips EM420 electron microscope.

### Western blotting analysis

Western blotting detected the protein level of MAP1LC3B, ATG12 and ATG5 in Caco-2 cells as described previously [23]. Briefly, cells were homogenized and washed with pre-cooling PBS and then lysed by the M-PER Mammalian Protein Extraction Reagent (Pierce, 78501, Thermo Scientific, Waltham, MA, USA). The protein assay kit (Pierce, 23227, Thermo Scientific) was used to measure the protein concentration. The lysates were separated by SDS-PAGE, and transferred to polyvinylidene difluoride membranes. Primary antibodies were diluted 1:1000. Membranes were developed using Supersignal® West Dura Duration substrate reagent (Thermo Scientific, 34080). Densitometric analysis on the western blot was done by Image Gauge software (Fujifilm, Maryland, USA). β-actin was used as an internal control, and the ratio of the intensity of interest protein to β-actin was calculated.

### ELISA

IL-8, TNF-α and IL-1β levels were measured in supernatants by DuoSet ELISA Development System (R&D, Minneapolis, USA) according to the manufacturer’s instructions.

### GFP-LC3 puncta formation assays

1×10^5^ Caco-2 cells were transfected with 150 nM GFP-MAP1LC3B plasmid at 37°C for 24 hours, and then changed with 2ml DMEM medium (Gibco, 11875–093) containing 10% fetal bovine serum. After *F*. *nucleatum* (OD_600nm_ = 1×10^7^ CFU/ml) infection 12 hours, cells were washed with 2ml PBS three times and fixed in 4% paraformaldehyde for 15 min. Confocal microscopy was performed with a Radiance 2000 laser scanning confocal microscope, followed by image analysis with LaserSharp 2000 software (Bio-Rad, San Francisco, CA). Images were acquired in a sequential scanning mode. According to methods for monitoring GFP-LC3 puncta formation assays [24], the average number of MAP1LC3B puncta per cell was determined. A minimum of 200 cells per sample was counted for triplicate samples per condition per experiment [25].

### Statistical analyses

Numerical data are expressed as mean ± standard error (SEM) from at least 3 separate experiments, with each being performed in triplicate. The differences were analyzed with the SPSS 13.0 software (SPSS, United States). The data between the three groups were assessed using one-way ANOVA. In addition, an analysis of *F*. *nucleatum* infection vs. control in different time was performed with *t-*test.

## Results

### *F*. *nucleatum* induces proinflammatory cytokines production in Caco-2 Cells

**Fig 1:**
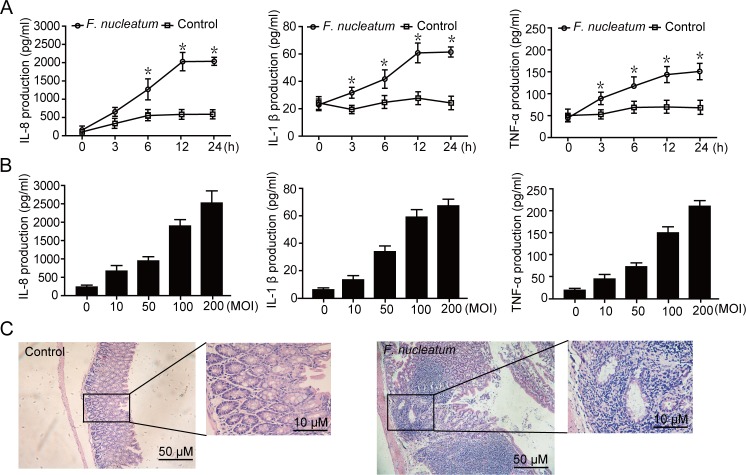
*F*. *nucleatum* induces proinflammatory cytokines production in Caco-2 Cells. (A and B) Caco-2 cells were infected by *F*. *nucleatum* for the indicated periods of time (A; MOI = 100:1) or at different MOIs (B; 10, 50, 100 and 200) for 12 h. Supernatants were assessed by ELISA for levels of IL-8, IL-1β and TNF-α. (C) Representative images of hematoxylin and eosin staining of colonic epithelium from C57BL/6 mice. Data are presented as the means ±SEM of three experiments. *P<0.05.

Accumulating number of papers reported that *F*. *nucleatum* infection could induce inflammation in different cells, including gingival epithelial cells and macrophage [26–27]. To clarify the role of *F*. *nucleatum* in the inflammation of intestinal tract, the protein levels of proinflammatory cytokines (IL-8, IL-1β and TNF-α), which are known to be involved in inflammation during *F*. *nucleatum* infection, was examined by ELISA assay. As shown in Fig 1A, levels of IL-8, IL-1β and TNF-α were significantly higher in Caco-2 cells infected with *F*. *nucleatum*. In addition, Caco-2 cells infected with *F*. *nucleatum* at increasing bacterial loads (MOI = 10, 50, 100, 200) produced greater amounts of IL-8, IL-1β and TNF-α (Fig 1B). Similar results were also obtained in the CW-2 cells (S1 Fig). To further investigate the inflammation by *F*. *nucleatum*, C57BL/6 mice was infused with the bacteria. As shown in Fig 1C, *F*. *nucleatum* significantly increased the inflammation of intestinal tract compared to control in C57BL/6 mice. Results demonstrate that *F*. *nucleatum* induces production of pro-inflammatory cytokines in Caco-2 cells.

### Expression of pro-inflammatory cytokine induced by *F*. *nucleatum* is dependent on ROS

**Fig 2:**
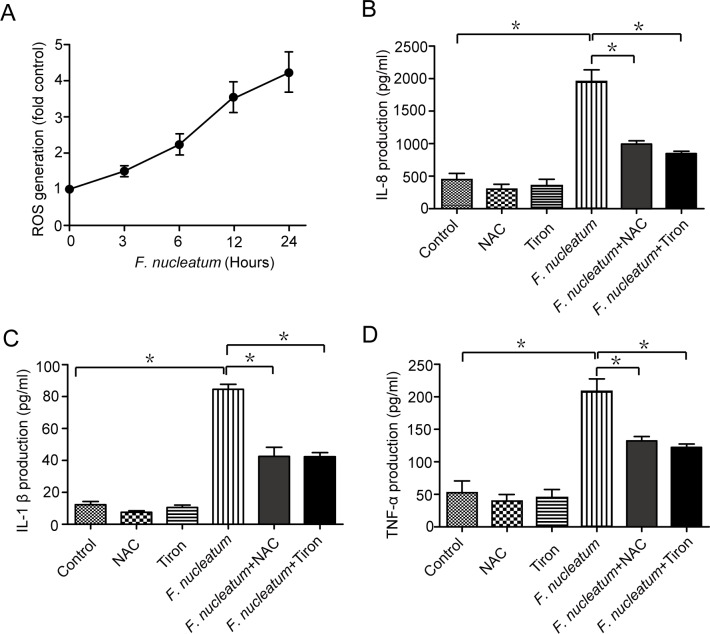
Inflammation induced by *F*. *nucleatum* is dependent on ROS. (A) Caco-2 cells were infected by *F*. *nucleatum* for the indicated periods of time (3, 6, 12, 24 hours). ROS generation was detected by DCFH-DA assay. (B, C and D) Following pretreatment with 1 mM Tiron or 10 mM N-acetyl-cysteine (NAC) for 6 hours, Caco-2 cells were infected with *F*. *nucleatum* (MOI = 100:1) for 12 hours. Supernatants of medium was assessed by ELISA for levels of IL-8, IL-1β and TNF-α. The data are presented as the means ±SEM of at least 3 independent experiments. *, *P*<0.05.

ROS generation is involved in regulating inflammation induced by bacteria infection [28–29]. To detect whether *F*. *nucleatum* could induce ROS generation, the intracellular levels of ROS were determined by DCFH-DA assay in Caco-2 cells following *F*. *nucleatum* infection. There was a time-dependent increase in the generation of ROS (Fig 2A). Similar results were also obtained in the CW-2 cells (S2A Fig). In addition, the ROS scavengers (NAC or Tiron), which could decrease ROS generation, suppressed the expression of IL-8, IL-1β and TNF-α (Fig 2B–2D, and S2B Fig). These data suggested that ROS had a critical role in *F*. *nucleatum*-induced inflammation.

### Autophagy is impaired in Caco-2 cells after *F*. *nucleatum* infection

**Fig 3:**
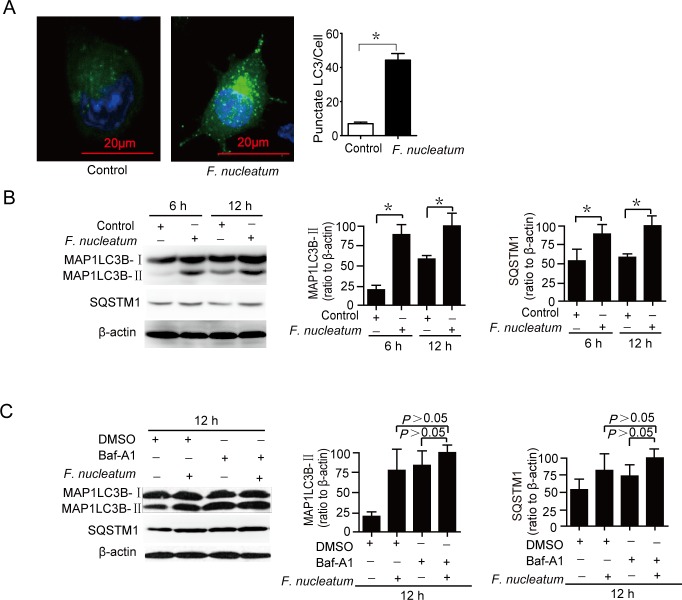
Autophagy is impaired in Caco-2 cells after *F*. *nucleatum* infection. (A) Caco-2 cells were transfected with a GFP-MAP1LC3B plasmid for 24 hours, and then infected with *F*. *nucleatum* for 12 hours. Confocal microscopy determined the number of GFP-MAP1LC3B puncta in each cell. (B) The protein level of SQSTM1 and the rate of MAP1LC3B-II to β-actin in Caco-2 cells were detected with western blot assay after *F*. *nucleatum* infection 12 hrs. (C) Caco-2 cells were infected with *F*. *nucleatum* for 12 hrs in the presence of Baf-A1 (10 nM), and western blot assay detected SQSTM1 and MAP1LC3B-II. Data are presented as the means ±SEM of three experiments. *P<0.05.

To illustrate whether *F*. *nucleatum* infection induced autophagy, GFP-MAPLC3B puncta formation assay was used to monitor autophagy. There was a significant increase in the percentage of cells with GFP-MAPLC3B puncta in *F*. *nucleatum*-infected Caco-2 cells after 12 hours compared with control cells (Fig 3A). To further confirm whether *F*. *nucleatum* induces autophagy in Caco-2 cells, the ratio of MAP1LC3B to β-actin was investigated by Western blot. There was a gradual increase in the ratio of MAP1LC3B-II to β-actin over time in cells infected with *F*. *nucleatum* compared to control cells, and another indicator SQSTM1 was also increased with *F*. *nucleatum* treatment (Fig 3B). This result indicated that autophagic flux might be impaired by *F*. *nucleatum* infection. To further confirm this result, we used Baf-A1(an autophagic flux inhibitor) to detect autophagic flux. Baf-A1 challenge did not result in the significant accumulation of MAP1LC3B-II and SQSTM1 in Caco-2 cells after 12 hrs (Fig 3C), suggesting that *F*. *nucleatum* impaired cellular autophagic flux in Caco-2 cells.

### Inhibition of autophagy enhances cytokines production and ROS generation induced by *F*. *nucleatum* infection

**Fig 4:**
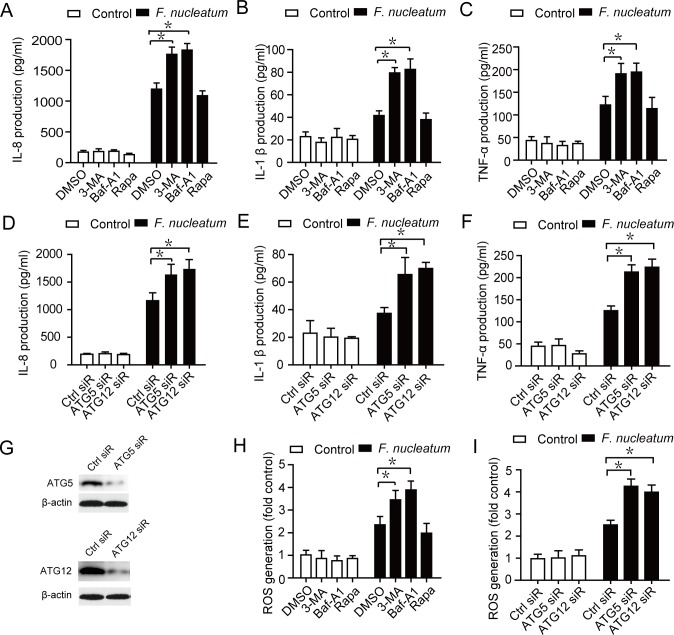
Inhibition of autophagy enhances cytokines production and ROS generation induced by *F*. *nucleatum* infection. (A, B, and C) After pretreatment of 0.1% DMSO, 3-MA (2mM), Baf-A1 (10 nM) or Rapa (100 nM), Caco-2 cells were infected with *F*. *nucleatum* (MOI = 100:1) for 12 hrs. Supernatants of medium were assessed by ELISA for levels of IL-8, IL-1β and TNF-α. (D, E and F) Production of IL-8, IL-1β and TNF-α in Caco-2 cells transfected with siRNA specific for ATG5 or ATG12 (50 nM) for 24h and infected with *F*. *nucleatum* (MOI = 100) for 12 hrs, as assessed by ELISA. (G) siRNAs targeting ATG5 or ATG12 (100 nM each) transfected Caco-2 cells for 24 hrs, and the protein levels were assayed by Western blotting. (H and I) After pretreatment of 0.1% DMSO, 3-MA (2mM), Baf-A1 (10 nM) or Rapa (100 nM), or transfected with siRNA specific for ATG5 or ATG12 (50 nM) for 24h, Caco-2 cells were infected with *F*. *nucleatum* (MOI = 100:1) for 12 hrs. ROS generation was detected by DCFH-DA assay. The data shown are the means ±SEM of three experiments. *, *P*<0.05.

Accumulating number of papers reported that autophagy plays a critical role in the inflammation. To further investigate the role of autophagy in the inflammation, we examined the production of IL-8, TNF-α and IL-1β by pretreatment with the autophagy inhibitors (3-MA or Baf-A1) or enhancer (Rapa, Rapamycin) upon *F*. *nucleatum* infection. Compared to the control groups, both 3-MA and Baf-A1 significantly increased the production of proinflammatory cytokines in Caco-2 cells infected by *F*. *nucleatum* (Fig 4A–4C). Moreover, to further address the possibility that the inhibition of autophagy is responsible for inflammation induced by *F*. *nucleatum*, we assessed the effects of ATG5 or ATG12 silencing by RNA interference (Fig 4G). The production of IL-8, IL-1β and TNF-α significantly increased in Caco-2 cells transfected with siRNA specific for ATG5 or ATG12 upon *F*. *nucleatum* infection (Fig 4D–4F). Similar results were also obtained in the CW-2 cells (S3A–S3C Fig). In addition, to confirm whether autophagy could regulate *F*. *nucleatum* -induced ROS generation, the intracellular levels of ROS was quantified by DCFH-DA assay in Caco-2 cells following treatment with 3-MA, Baf-A1, Rapa, *F*. *nucleatum*, or a combination. There was a significant increase in the generation of ROS in cells treated with *F*. *nucleatum* combination with 3- MA or Baf-A1 compared with cells treated with *F*. *nucleatum* alone (Fig 4H). The production of ROS in ATG5 or ATG12 siRNA group was significantly higher than that of control siRNA group upon infection with the *F*. *nucleatum* (Fig 4I, and S3D Fig). These results indicated that autophagy has an important role in the inflammation during *F*. *nucleatum* infection.

## Discussion

**Fig 5:**
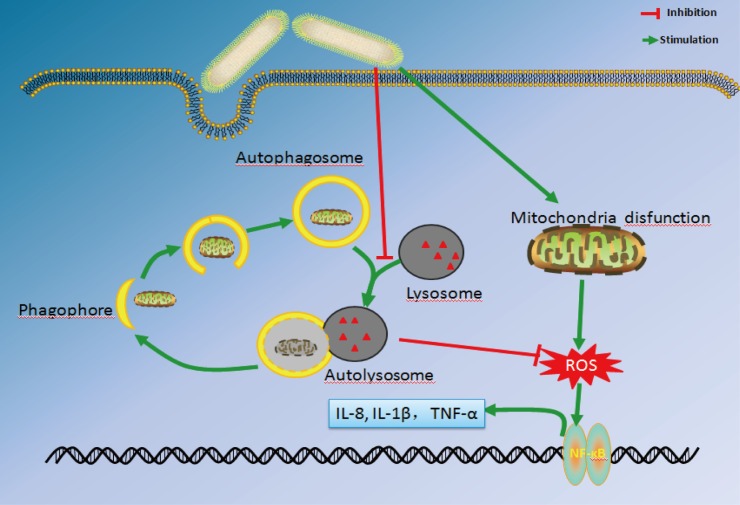
Schematic of the proposed mechanism of *Fusobacterium nucleatum*-induced impairment of autophagic flux enhancing the expression of proinflammatory cytokines via ROS in Caco-2 Cells (see text for details).

Based on the present results, it is firstly proposed that *Fusobacterium nucleatum*-induced impairment of autophagic flux enhances the expression of proinflammatory cytokines via ROS in Caco-2 Cells (Fig 5). The novel model is supported by the following data: i) *F*. *nucleatum* induces proinflammatory cytokines production in Caco-2 Cells, ii) Inflammation induced by *F*. *nucleatum* is dependent on ROS, iii) Autophagy is impaired in Caco-2 cells after *F*. *nucleatum* infection, and iv) Inhibition of autophagy enhances cytokines production and ROS generation induced by *F*. *nucleatum* infection. Importantly, our findings shed light on novel mechanism of *F*. *nucleatum*-induced inflammation.

As quality control of cellular milieu, autophagy plays an important role for provoking a protective response during infection [30]. However, a lot of pathogens can subvert autophagy to promote inflammation, cell death, and genetic instability [30–31]. For example, VacA induces autophagosome formation during *H*. *pylori* infection in vitro[32], and that VacA could disrupt autophagy to promote the infection in human [33]. In the present study, we discovered that *F*. *nucleatum* could induce autophagosome generation, but suppress its maturation. Baf-A1 challenge did not result in the significant accumulation of MAP1LC3B-II and SQSTM1 in Caco-2 cells after 24 hrs (Fig 3C). We also assessed whether inflammation induced by *F*. *nucleatum* was regulated by autophagy inhibitors 3-MA or Baf-A1, and siRNA targeting ATG5 and ATG12. Remarkably, both 3-MA or Baf-A1 treatment and the siRNA increased the amount of *F*. *nucleatum*-induced inflammation. These results indicate that the impairment of autophagic flux directly promotes the inflammation induced by *F*. *nucleatum*. However, it is currently unclear how *F*. *nucleatum* impair autophagic flux, which study is currently in progress.

Previous study reported that both the ROS and autophagy pathways are new mechanisms of regulating inflammation [34]. Additionally, ROS plays an important role in autophagy generation in organisms ranging from yeast to mammals [35]. However, it remain unclear whether disruption of autophagy or generation of ROS plays a critical role in the inflammation induced by *F*. *nucleatum*. In the study, autophagy was impaired by *F*. *nucleatum* as a inflammatory response in intestinal epithelial cells, which response was regulated by ROS. Moreover, ROS scavengers NAC or Tiron could suppress the expression of IL-8, IL-1β and TNF-α (Fig 2B–2D), suggested that ROS could mediated *F*. *nucleatum*-induced inflammation. In addition, there were significant increases in the generation of ROS in cells treated with *F*. *nucleatum* combination with 3-MA or Baf-A1 compared with cells treated with *F*. *nucleatum* alone (Fig 4H). The production of ROS in ATG5 or ATG12 siRNA group was significantly higher than that of control siRNA group upon infection with the *F*. *nucleatum* (Fig 4I). Taken together, the data suggested that *F*. *nucleatum*-mediated inhibition of autophagic process leads to accumulation of ROS, resulting in proinflammatory cytokine production.

In summary, our results show that *F*. *nucleatum* infection impairs autophagic flux within intestine epithelial cells, leading to ROS accumulation and proinflammatory cytokine production. Although the specific mechanism of autophagic flux impairment by *F*. *nucleatum* infection remains to be determined by further experiments, our studies establish the basis to evaluate the role of autophagy during *F*. *nucleatum* infection in the future.

## Supporting Information

S1 Fig *F*. *nucleatum* induces proinflammatory cytokines production in CW-2 Cells. (A) CW-2 cells were infected with *F*. *nucleatum* for 24 h. Supernatants were assessed by ELISA for levels of IL-8, IL-1β and TNF-α. Data are presented as the means±SEM of three experiments. *P<0.05, ** *P*<0.01.
(TIF)

S2 Fig Inflammation induced by *F*. *nucleatum* is dependent on ROS. (A) CW-2 cells were infected by *F*. *nucleatum* for the indicated periods of time (3, 6, 12, 24 hours). ROS generation was detected by DCFH-DA assay. (B) Following pretreatment with 1 mM Tiron or 10 mM N-acetyl-cysteine (NAC) for 6 hours, CW-2 cells were infected with *F*. *nucleatum* (MOI = 100:1) for 12 hours. Supernatants of medium was assessed by ELISA for levels of IL-8. (C) The effects of Tiron or NAC on the cytotoxicity of *F*. *nucleatum* in Caco-2 cells. After pretreatment with 1 mM Tiron or 10 mM NAC, Caco-2 cells were treated with *F*. *nucleatum* for 12 h. The percentage of dead cells was determined using the cell death assay (PI staining) or the cell viability assay (MTT). The data are presented as the means ±SEM of at least 3 independent experiments. *, *P*<0.05.
(TIF)

S3 Fig Inhibition of autophagy enhances cytokines production and ROS generation induced by *F*. *nucleatum* infection. (A, B and C) Production of IL-8, IL-1β and TNF-α in CW-2 cells transfected with siRNA specific for ATG5 or ATG12 (50 nM) for 24h and infected with *F*. *nucleatum* (MOI = 100) for 12 hrs, as assessed by ELISA. (D) After transfected with siRNA specific for ATG5 or ATG12 (50 nM) for 24h, CW-2 cells were infected with *F*. *nucleatum* (MOI = 100:1) for 12 hrs. ROS generation was detected by DCFH-DA assay. The data shown are the means ±SEM of three experiments. *, *P*<0.05.
(TIF)
