# Supplementary figures and images for: Fusobacterium nucleatum-Induced Impairment of Autophagic Flux Enhances the Expression of Proinflammatory Cytokines via ROS in Caco-2 Cells
Source: PLoS One. 2016 Nov 9;11(11):e0165701. doi: 10.1371/journal.pone.0165701 (PMC5102440; doi:10.1371/journal.pone.0165701)

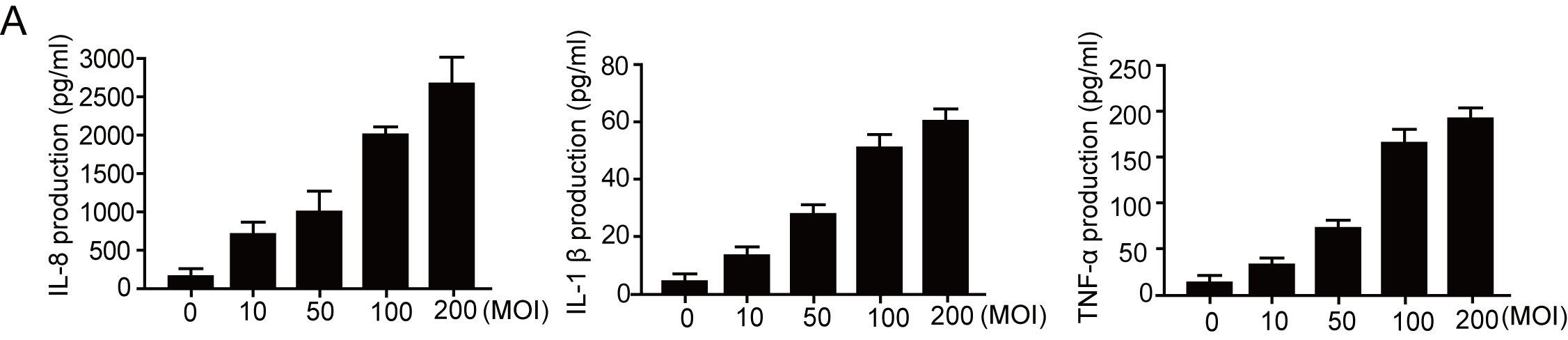

Supplement: S1 Fig — (A) CW-2 cells were infected with F. nucleatum for 24 h. Supernatants were assessed by ELISA for levels of IL-8, IL-1β and TNF-α. Data are presented as the means±SEM of three experiments. *P<0.05, ** P<0.01. (TIF) [file pone.0165701.s001.tif]

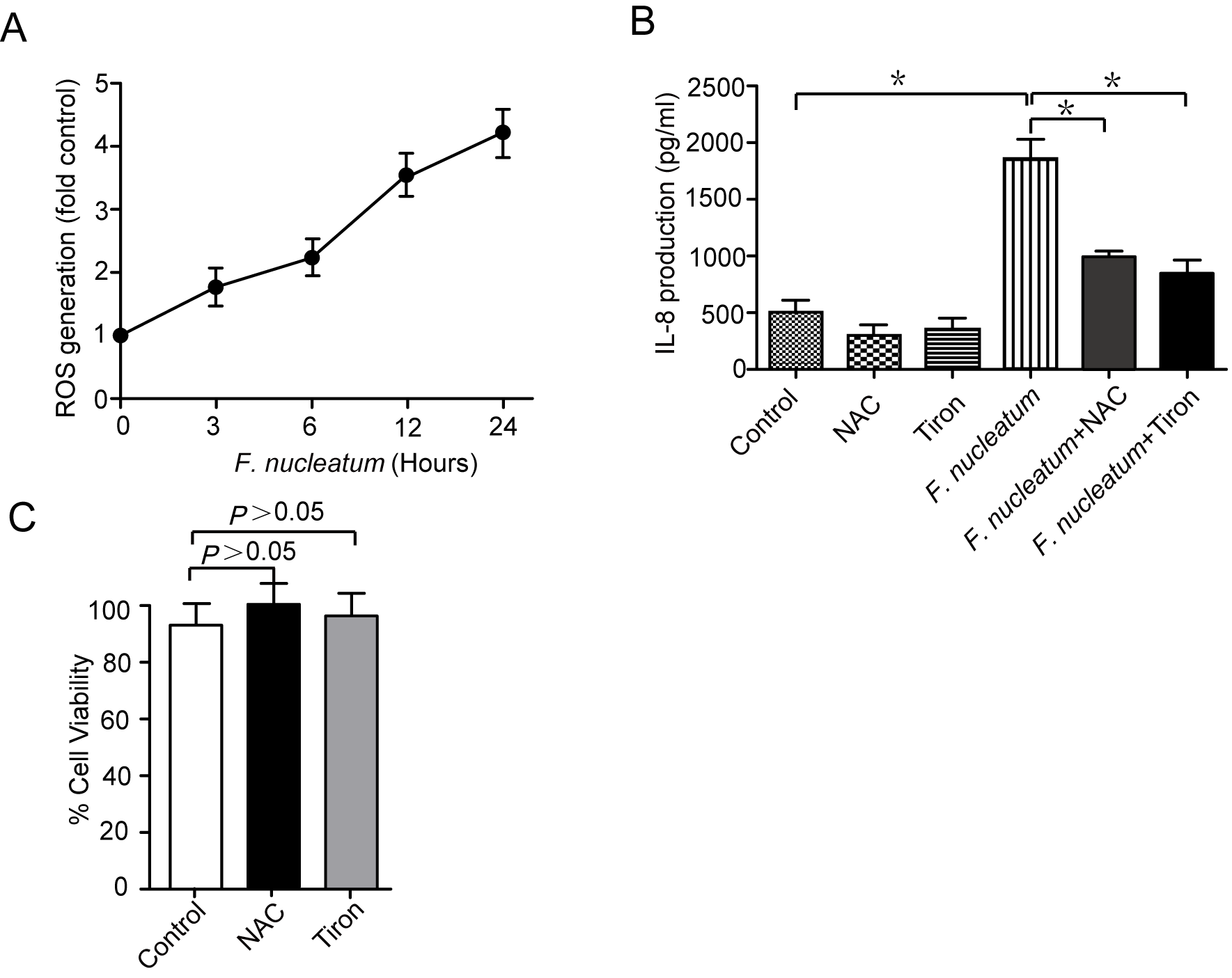

Supplement: S2 Fig — (A) CW-2 cells were infected by F. nucleatum for the indicated periods of time (3, 6, 12, 24 hours). ROS generation was detected by DCFH-DA assay. (B) Following pretreatment with 1 mM Tiron or 10 mM N-acetyl-cysteine (NAC) for 6 hours, CW-2 cells were infected with F. nucleatum (MOI = 100:1) for 12 hours. Supernatants of medium was assessed by ELISA for levels of IL-8. (C) The effects of Tiron or NAC on the cytotoxicity of F. nucleatum in Caco-2 cells. After pretreatment with 1 mM Tiron or 10 mM NAC, Caco-2 cells were treated with F. nucleatum for 12 h. The percentage of dead cells was determined using the cell death assay (PI staining) or the cell viability assay (MTT). The data are presented as the means ±SEM of at least 3 independent experiments. *, P<0.05. (TIF) [file pone.0165701.s002.tif]

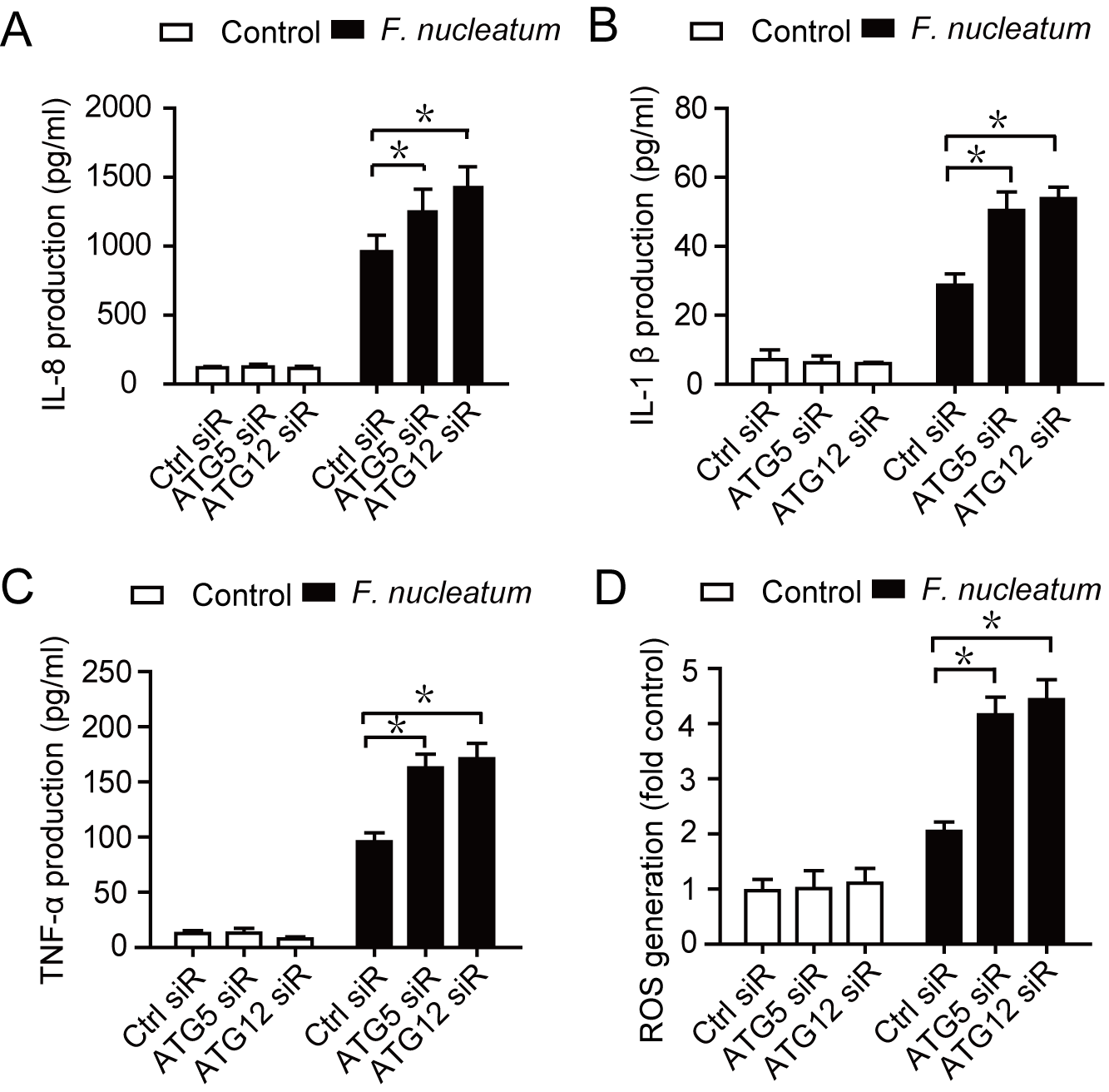

Supplement: S3 Fig — (A, B and C) Production of IL-8, IL-1β and TNF-α in CW-2 cells transfected with siRNA specific for ATG5 or ATG12 (50 nM) for 24h and infected with F. nucleatum (MOI = 100) for 12 hrs, as assessed by ELISA. (D) After transfected with siRNA specific for ATG5 or ATG12 (50 nM) for 24h, CW-2 cells were infected with F. nucleatum (MOI = 100:1) for 12 hrs. ROS generation was detected by DCFH-DA assay. The data shown are the means ±SEM of three experiments. *, P<0.05. (TIF) [file pone.0165701.s003.tif]
